# Supplementary material for: Short-Term Effects of PM10, NO2, SO2 and O3 on Cardio-Respiratory Mortality in Cape Town, South Africa, 2006–2015
Source: Int J Environ Res Public Health. 2022 Jun 30;19(13):8078. doi: 10.3390/ijerph19138078 (PMC9265394; doi:10.3390/ijerph19138078)
Supplement: Supplementary file 1 [file ijerph-19-08078-s001.zip › ijerph-1796390-supplementary.pdf]

Short-Term Effects of PM_{10}, NO_2, SO_2 and O_3 on Cardio-Respiratory Mortality in Cape Town, South Africa, 2006–2015

Temitope Christina Adebayo-Ojo [1,2,*], Janine Wichmann [3], Oluwaseyi Olalekan Arowosegbe [1,2], Nicole Probst-Hensch [1,2], Christian Schindler [1,2] and Nino Künzli [1,2]

[1] Department of Epidemiology and Public Health, Swiss Tropical and Public Health Institute, Kreuzstrasse 2, Allschwil, 4123 Basel, Switzerland; oluwaseyiolalekan.arowosegbe@swisstph.ch (O.O.A.); nicole.probst@swisstph.ch (N.P.-H.); christian.schindler@swisstph.ch (C.S.); nino.kuenzli@swisstph.ch (N.K.)

[2] Faculty of Medicine, University of Basel, 4056 Basel, Switzerland

[3] Faculty of Health Sciences, School of Health Systems and Public Health, University of Pretoria, Pretoria 0002, South Africa; janine.wichmann@up.ac.za

* Correspondence: temitope.adebayo@swisstph.ch

Time-Series Analysis Results

This document tabulates the correlations among environmental variables and provides estimates for the two-day means of the four pollutants by age group, sex and season, and for the lag structures of effects over 21 days by age group.

Table S1. Temporal spearman rank correlation coefficients among daily mean concentrations of ambient air pollutants and meteorological variables during the year and by seasons.

	PM_{10}	NO_2	SO_2	O_3	Temperature	Humidity
PM_{10}	1	0.38	0.27	0.12	0.17	-0.25
NO_2		1	0.4	0.06	-0.35	0.07
SO_2			1	-0.11	-0.12	-0.01
O_3				1	-0.13	0.01
Temperature					1	-0.41
Humidity						1
Warm /dry season		November - March				
PM_{10}	1	0.29	0.28	0.23	0.33	-0.27
NO_2		1	0.32	0.09	-0.22	-0.03
SO_2			1	-0.07	0.01	-0.07
O_3				1	-0.14	-0.02
Temperature					1	-0.26
Humidity						1
Cold / wet season		April – October				
PM_{10}	1	0.54	0.27	-0.09	0.03	-0.3
NO_2		1	0.46	-0.15	-0.11	-0.15
SO_2			1	-0.25	-0.05	-0.11
O_3				1	0.01	-0.01
Temperature					1	-0.02
Humidity						1

The relative risk presented in the following table were estimated from Quasi-Poisson regression models of respiratory and cardiovascular disease deaths, adjusting for time trends and seasonal variation, day of the week, public holiday, and meteorological factors including temperature and relative humidity.

Table S2. Single and multiple pollutant model adjusted relative risk (RR) for an interquartile range increase in the two-day moving average of PM_{10} concentrations and mortality due to cardiovascular and respiratory diseases in all ages, age groups and sex, Cape Town, South Africa, 1 January 2006–31 December 2015.

		Respiratory disease			Cardiovascular disease		
	Pollutant IQR (16 μg/m³)	Relative risk	95% Confidence interval		Relative risk	95% Confidence interval	
			Lower interval	Upper interval		Lower interval	Upper interval
All ages and sex	PM_{10}	1.003	0.978	1.029	1.024	1.009	1.039
Age 15 - 64		1.003	0.969	1.039	1.011	0.986	1.036
Age >65		1.003	0.969	1.038	1.033	1.014	1.052
Female		1.009	0.973	1.047	1.032	1.012	1.053
Male		1	0.967	1.033	1.015	0.994	1.037
All ages and sex	PM_{10} NO_2	0.98	0.951	1.011	1.019	1	1.037
Age 15 - 64		0.975	0.934	1.018	1.004	0.974	1.035
Age >65		0.985	0.944	1.027	1.028	1.005	1.052
Female		0.98	0.937	1.025	1.026	1.001	1.051
Male		0.981	0.942	1.021	1.011	0.986	1.038
All ages and sex	PM_{10} SO_2	0.999	0.973	1.027	1.022	1.006	1.039
Age 15 - 64		0.996	0.96	1.034	1.01	0.984	1.037
Age >65		1.003	0.966	1.04	1.03	1.01	1.051
Female		1.003	0.965	1.043	1.034	1.012	1.056
Male		0.997	0.963	1.033	1.01	0.988	1.033
All ages and sex	PM_{10} O_3	0.999	0.968	1.031	1.028	1.01	1.047
Age 15 - 64		1.005	0.963	1.05	1.008	0.979	1.039
Age >65		0.994	0.952	1.038	1.042	1.018	1.066
Female		0.99	0.946	1.037	1.044	1.018	1.07
Male		1.005	0.964	1.047	1.013	0.987	1.04
All ages and sex	PM_{10} NO_2 SO_2	0.98	0.95	1.012	1.018	0.999	1.037
Age 15 - 64		0.974	0.932	1.018	1.003	0.972	1.035

Age >65		0.986	0.944	1.03	1.028	1.004	1.052
Female		0.975	0.931	1.021	1.028	1.002	1.055
Male		0.984	0.944	1.026	1.007	0.98	1.034
All ages and sex	PM_{10} SO_2 O_3	0.996	0.964	1.03	1.03	1.01	1.05
Age 15 - 64		1.001	0.956	1.048	1.006	0.975	1.038
Age >65		0.994	0.949	1.04	1.046	1.021	1.072
Female		0.988	0.941	1.037	1.048	1.021	1.076
Male		1.002	0.959	1.046	1.013	0.985	1.041
All ages and sex	PM_{10} NO_2 O_3	0.984	0.948	1.023	1.019	0.996	1.042
Age 15 - 64		0.996	0.944	1.05	0.989	0.953	1.026
Age >65		0.973	0.923	1.026	1.038	1.009	1.067
Female		0.983	0.929	1.04	1.03	0.999	1.062
Male		0.984	0.936	1.034	1.007	0.975	1.039
All ages and sex	PM_{10} NO_2 SO_2 O_3	0.985	0.947	1.024	1.02	0.997	1.044
Age 15 - 64		0.996	0.943	1.052	0.984	0.947	1.022
Age >65		0.974	0.922	1.029	1.043	1.013	1.074
Female		0.979	0.924	1.038	1.033	1.001	1.067
Male		0.987	0.937	1.039	1.006	0.973	1.039

Table S3. Single and multiple pollutant model adjusted relative risk (RR) for an interquartile range increase in the two-day moving average of NO_2 concentrations and mortality due to cardiovascular and respiratory diseases in all ages, age groups and sex, Cape Town, South Africa, 1 January 2006 - 31 December 2015.

		Respiratory disease			Cardiovascular disease		
	Pollutant IQR (11 μg/m³)	Relative risk	95% Confidence interval		Relative risk	95% Confidence interval	
			Lower interval	Upper interval		Lower interval	Upper interval
All ages and sex	NO_2	1.045	1.014	1.076	1.022	1.004	1.041
Age 15 - 64		1.041	0.997	1.085	1.01	0.981	1.041
Age >65		1.049	1.007	1.093	1.031	1.008	1.054
Female		1.049	1.004	1.096	1.026	1.001	1.051
Male		1.042	1.002	1.083	1.019	0.993	1.045
All ages and sex	NO_2 PM_{10}	1.058	1.022	1.095	1.01	0.989	1.031
Age 15 - 64		1.055	1.004	1.108	1.007	0.973	1.042
Age >65		1.061	1.012	1.113	1.013	0.987	1.04
Female		1.063	1.01	1.119	1.012	0.983	1.041
Male		1.054	1.008	1.103	1.009	0.979	1.039
All ages and sex	NO_2 SO_2	1.043	1.011	1.077	1.019	1	1.039
Age 15 - 64		1.042	0.996	1.09	1.007	0.976	1.039
Age >65		1.046	1.001	1.092	1.028	1.004	1.053
Female		1.047	0.999	1.097	1.025	0.999	1.052
Male		1.043	1	1.088	1.013	0.987	1.041
All ages and sex	NO_2 O_3	1.031	0.992	1.07	1.031	1.009	1.054
Age 15 - 64		1.018	0.965	1.074	1.026	0.989	1.063
Age >65		1.042	0.99	1.098	1.038	1.01	1.068
Female		1.003	0.948	1.061	1.039	1.008	1.071
Male		1.053	1.002	1.106	1.027	0.995	1.06
All ages and sex	NO_2 PM_{10} O_3	1.041	0.996	1.088	1.017	0.991	1.044
Age 15 - 64		1.018	0.956	1.084	1.03	0.988	1.075

Age >65		1.063	1.001	1.129	1.013	0.98	1.046
Female		1.015	0.95	1.084	1.021	0.985	1.058
Male		1.063	1.003	1.126	1.016	0.979	1.055
All ages and sex	NO_2 PM_{10} SO_2	1.055	1.018	1.093	1.008	0.987	1.03
Age 15 - 64		1.054	1.002	1.109	1.004	0.97	1.04
Age >65		1.056	1.005	1.109	1.012	0.986	1.04
Female		1.063	1.008	1.12	1.011	0.982	1.041
Male		1.052	1.003	1.102	1.006	0.976	1.036
All ages and sex	NO_2 SO_2 O_3	1.031	0.991	1.074	1.035	1.011	1.059
Age 15 - 64		1.018	0.961	1.078	1.025	0.987	1.065
Age >65		1.043	0.987	1.102	1.044	1.014	1.075
Female		0.999	0.941	1.061	1.045	1.012	1.078
Male		1.059	1.005	1.116	1.027	0.994	1.062
All ages and sex	NO_2 PM_{10} SO_2 O_3	1.04	0.994	1.088	1.021	0.994	1.049
Age 15 - 64		1.017	0.954	1.084	1.031	0.988	1.077
Age >65		1.061	0.997	1.129	1.019	0.985	1.054
Female		1.012	0.946	1.082	1.028	0.991	1.066
Male		1.065	1.004	1.13	1.018	0.98	1.057

Table S4. Single and multiple pollutant model adjusted relative risk (RR) for an interquartile range increase in the two-day moving average of SO_2 concentrations and mortality due to cardiovascular and respiratory diseases in all ages, age groups and sex, Cape Town, South Africa, 1 January 2006 - 31 December 2015.

		Respiratory disease			Cardiovascular disease		
	Pollutant IQR (6 μg/m³)	Relative risk	95% Confidence interval		Relative risk	95% Confidence interval	
			Lower interval	Upper interval		Lower interval	Upper interval
All ages and sex	SO_2	1.013	0.99	1.036	1.014	1	1.028
Age 15 - 64		1.01	0.979	1.042	1.01	0.988	1.032
Age >65		1.016	0.985	1.047	1.017	0.999	1.034
Female		1.021	0.988	1.055	1.007	0.989	1.026
Male		1.005	0.976	1.035	1.022	1.003	1.041
All ages and sex	SO_2 PM_{10}	1.013	0.99	1.037	1.008	0.993	1.022
Age 15 - 64		1.011	0.978	1.045	1.007	0.984	1.03
Age >65		1.015	0.983	1.048	1.008	0.99	1.026
Female		1.02	0.985	1.055	0.998	0.979	1.018
Male		1.007	0.976	1.039	1.019	0.998	1.039
All ages and sex	SO_2 NO_2	1.002	0.977	1.027	1.01	0.995	1.025
Age 15 - 64		0.997	0.963	1.033	1.012	0.988	1.036
Age >65		1.007	0.973	1.042	1.008	0.989	1.027
Female		1.013	0.976	1.05	1.003	0.984	1.024
Male		0.992	0.96	1.025	1.017	0.997	1.039
All ages and sex	SO_2 O_3	1.002	0.972	1.033	1.007	0.988	1.026
Age 15 - 64		1.007	0.965	1.05	0.999	0.97	1.03
Age >65		0.999	0.958	1.043	1.012	0.988	1.036
Female		1.004	0.959	1.05	1.003	0.978	1.028
Male		1	0.961	1.04	1.012	0.986	1.039
All ages and sex	SO_2 PM_{10} O_3	1.004	0.973	1.037	0.996	0.977	1.016
Age 15 - 64		1.007	0.963	1.053	0.999	0.968	1.03

Age >65		1.003	0.959	1.048	0.995	0.971	1.02
Female		1.008	0.961	1.058	0.987	0.961	1.014
Male		1.001	0.96	1.044	1.007	0.98	1.036
All ages and sex	SO_2 PM_{10} NO_2	1.005	0.98	1.031	1.007	0.992	1.022
Age 15 - 64		1.001	0.966	1.038	1.012	0.988	1.038
Age >65		1.009	0.975	1.045	1.003	0.984	1.022
Female		1.016	0.979	1.055	0.998	0.978	1.019
Male		0.996	0.963	1.03	1.017	0.996	1.039
All ages and sex	SO_2 NO_2 O_3	0.995	0.962	1.03	0.997	0.977	1.018
Age 15 - 64		0.996	0.95	1.045	1.004	0.971	1.037
Age >65		0.996	0.95	1.045	0.993	0.967	1.019
Female		1.011	0.96	1.064	0.991	0.964	1.019
Male		0.983	0.94	1.028	1.004	0.975	1.034
All ages and sex	SO_2 PM_{10} NO_2 O_3	1	0.965	1.036	0.993	0.972	1.014
Age 15 - 64		0.998	0.95	1.049	1.009	0.975	1.044
Age >65		1.002	0.954	1.053	0.982	0.956	1.009
Female		1.015	0.963	1.071	0.984	0.956	1.013
Male		0.988	0.944	1.034	1.003	0.974	1.034

Table S5. Single and multiple pollutant model adjusted relative risk (RR) for an interquartile range increase in the two-day moving average of O_3 concentrations and mortality due to cardiovascular and respiratory diseases in all ages, age groups and sex, Cape Town, South Africa, 1 January 2006 - 31 December 2015.

		Respiratory disease			Cardiovascular disease		
	Pollutant IQR (16 μg/m³)	Relative risk	95% Confidence interval		Relative risk	95% Confidence interval	
			Lower interval	Upper interval		Lower interval	Upper interval
All ages and sex	O_3	1.006	0.965	1.049	1.025	1.002	1.048
Age 15 - 64		1.007	0.951	1.067	1.019	0.982	1.057
Age >65		1.004	0.948	1.063	1.029	1	1.059
Female		0.949	0.894	1.008	1.018	0.987	1.05
Male		1.054	0.998	1.113	1.035	1.002	1.069
All ages and sex	O_3 PM_{10}	1.002	0.961	1.046	1.03	1.006	1.054
Age 15 - 64		1.005	0.947	1.066	1.019	0.982	1.058
Age >65		1	0.943	1.06	1.037	1.008	1.067
Female		0.944	0.887	1.005	1.025	0.993	1.057
Male		1.051	0.993	1.112	1.038	1.004	1.072
All ages and sex	O_3 NO_2	0.998	0.955	1.044	1.025	1.001	1.05
Age 15 - 64		0.989	0.929	1.053	1.02	0.981	1.06
Age >65		1.006	0.945	1.07	1.031	1.001	1.063
Female		0.932	0.873	0.995	1.024	0.991	1.058
Male		1.054	0.994	1.118	1.03	0.996	1.066
All ages and sex	O_3 SO_2	1.006	0.965	1.049	1.022	0.998	1.046
Age 15 - 64		1.005	0.948	1.065	1.014	0.977	1.053
Age >65		1.006	0.949	1.065	1.027	0.998	1.058
Female		0.952	0.896	1.012	1.015	0.984	1.047
Male		1.051	0.995	1.111	1.032	0.998	1.066
All ages and sex	O_3 PM_{10} SO_2	1.002	0.96	1.046	1.027	1.003	1.051
Age 15 - 64		1.001	0.943	1.063	1.014	0.977	1.053

Age >65		1.002	0.944	1.063	1.036	1.006	1.066
Female		0.947	0.889	1.008	1.022	0.99	1.055
Male		1.047	0.989	1.108	1.034	1.001	1.069
All ages and sex	O_3 PM_{10} NO_2	0.993	0.949	1.038	1.027	1.002	1.052
Age 15 - 64		0.983	0.923	1.047	1.018	0.979	1.059
Age >65		0.999	0.939	1.064	1.035	1.004	1.066
Female		0.928	0.868	0.991	1.026	0.993	1.061
Male		1.046	0.985	1.111	1.03	0.996	1.066
All ages and sex	O_3 SO_2 NO_2	0.999	0.955	1.044	1.025	1.001	1.05
Age 15 - 64		0.989	0.928	1.052	1.018	0.979	1.059
Age >65		1.006	0.946	1.071	1.032	1.001	1.064
Female		0.934	0.875	0.997	1.022	0.989	1.056
Male		1.054	0.993	1.118	1.032	0.998	1.068
All ages and sex	O_3 PM_{10} NO_2 SO_2	0.993	0.949	1.039	1.027	1.003	1.052
Age 15 - 64		0.983	0.922	1.047	1.016	0.977	1.057
Age >65		1	0.939	1.065	1.036	1.005	1.068
Female		0.928	0.869	0.992	1.025	0.992	1.059
Male		1.046	0.985	1.111	1.033	0.998	1.069

Table S6. Single and multiple pollutant model adjusted relative risk (RR) for 10 μg/m^3 increase in the two-day moving average of PM_{10} concentrations and mortality due to cardiovascular and respiratory diseases in all ages, age groups and sex, Cape Town, South Africa, 1 January 2006 - 31 December 2015.

		Respiratory disease			Cardiovascular disease		
	Pollutant (10 μg/m^3)	Relative risk	95% Confidence interval		Relative risk	95% Confidence interval	
			Lower interval	Upper interval		Lower interval	Upper interval
All ages and sex	PM_{10}	1.002	0.987	1.017	1.015	1.005	1.024
Age 15 - 64		1.002	0.981	1.024	1.007	0.992	1.022
Age >65		1.002	0.981	1.023	1.02	1.008	1.032
Female		1.006	0.983	1.028	1.02	1.007	1.032
Male		1	0.98	1.02	1.009	0.996	1.022
All ages and sex	PM_{10} NO_2	0.988	0.97	1.007	1.011	1	1.023
Age 15 - 64		0.985	0.959	1.011	1.002	0.984	1.021
Age >65		0.991	0.965	1.017	1.017	1.003	1.031
Female		0.988	0.961	1.015	1.016	1	1.031
Male		0.988	0.964	1.013	1.007	0.991	1.023
All ages and sex	PM_{10} SO_2	1	0.983	1.016	1.014	1.004	1.023
Age 15 - 64		0.998	0.975	1.021	1.006	0.99	1.023
Age >65		1.002	0.979	1.024	1.018	1.006	1.031
Female		1.002	0.979	1.026	1.021	1.007	1.034
Male		0.998	0.977	1.02	1.006	0.992	1.02
All ages and sex	PM_{10} O_3	0.999	0.98	1.019	1.017	1.006	1.029
Age 15 - 64		1.003	0.977	1.03	1.005	0.987	1.024
Age >65		0.996	0.97	1.023	1.025	1.011	1.04
Female		0.994	0.967	1.022	1.027	1.011	1.042
Male		1.003	0.978	1.028	1.008	0.992	1.024
All ages and sex	PM_{10} NO_2 SO_2	0.988	0.969	1.007	1.011	0.999	1.023
Age 15 - 64		0.984	0.958	1.011	1.002	0.983	1.021

Age >65		0.992	0.966	1.018	1.017	1.002	1.032
Female		0.985	0.957	1.013	1.017	1.001	1.033
Male		0.99	0.966	1.016	1.004	0.988	1.021
All ages and sex	PM_{10} SO_2 O_3	0.998	0.978	1.018	1.018	1.006	1.03
Age 15 - 64		1	0.973	1.029	1.004	0.984	1.023
Age >65		0.996	0.969	1.024	1.028	1.013	1.043
Female		0.992	0.963	1.022	1.029	1.012	1.046
Male		1.001	0.975	1.028	1.008	0.991	1.025
All ages and sex	PM_{10} NO_2 O_3	0.99	0.968	1.014	1.012	0.998	1.026
Age 15 - 64		0.998	0.966	1.031	0.993	0.971	1.016
Age >65		0.984	0.952	1.016	1.023	1.005	1.041
Female		0.99	0.956	1.025	1.018	0.999	1.038
Male		0.99	0.96	1.021	1.004	0.985	1.024
All ages and sex	PM_{10} NO_2 SO_2 O_3	0.991	0.967	1.015	1.012	0.998	1.027
Age 15 - 64		0.997	0.965	1.031	0.99	0.967	1.013
Age >65		0.984	0.952	1.018	1.026	1.008	1.045
Female		0.987	0.953	1.023	1.02	1.001	1.04
Male		0.992	0.961	1.024	1.003	0.983	1.024

Table S7. Single and multiple pollutant model adjusted relative risk (RR) for 10 μg/m³ increase in the two-day moving average of NO_2 concentrations and mortality due to cardiovascular and respiratory diseases in all ages, age groups and sex, Cape Town, South Africa, 1 January 2006 - 31 December 2015.

		Respiratory disease			Cardiovascular disease		
	Pollutant (10 μg/m³)	Relative risk	95% Confidence interval		Relative risk	95% Confidence interval	
			Lower interval	Upper interval		Lower interval	Upper interval
All ages and sex	NO_2	1.042	1.013	1.072	1.021	1.004	1.038
Age 15 - 64		1.038	0.998	1.08	1.01	0.982	1.038
Age >65		1.046	1.007	1.087	1.029	1.007	1.051
Female		1.026	1.002	1.051	1.024	1.001	1.048
Male		1.039	1.002	1.078	1.018	0.994	1.042
All ages and sex	NO_2 PM_{10}	1.054	1.02	1.089	1.009	0.99	1.029
Age 15 - 64		1.051	1.004	1.101	1.006	0.974	1.039
Age >65		1.057	1.011	1.106	1.012	0.988	1.037
Female		1.059	1.009	1.111	1.011	0.984	1.038
Male		1.051	1.007	1.097	1.008	0.981	1.036
All ages and sex	NO_2 SO_2	1.041	1.01	1.072	1.018	1	1.036
Age 15 - 64		1.039	0.996	1.084	1.007	0.978	1.037
Age >65		1.043	1.001	1.086	1.026	1.004	1.05
Female		1.044	0.999	1.091	1.023	0.999	1.048
Male		1.04	1	1.082	1.013	0.988	1.038
All ages and sex	NO_2 O_3	1.029	0.993	1.066	1.029	1.008	1.051
Age 15 - 64		1.017	0.967	1.069	1.024	0.99	1.059
Age >65		1.04	0.99	1.091	1.036	1.009	1.063
Female		1.003	0.951	1.057	1.036	1.007	1.066
Male		1.049	1.002	1.099	1.025	0.995	1.056
All ages and sex	NO_2 PM_{10} O_3	1.039	0.996	1.082	1.016	0.992	1.041
Age 15 - 64		1.017	0.959	1.078	1.029	0.988	1.07

Age >65		1.059	1.001	1.121	1.012	0.981	1.043
Female		1.014	0.953	1.079	1.02	0.986	1.055
Male		1.059	1.003	1.118	1.015	0.981	1.051
All ages and sex	NO_2 PM_{10} SO_2	1.051	1.017	1.087	1.008	0.988	1.028
Age 15 - 64		1.051	1.002	1.102	1.004	0.971	1.037
Age >65		1.052	1.005	1.102	1.011	0.986	1.037
Female		1.059	1.008	1.112	1.011	0.984	1.038
Male		1.048	1.003	1.095	1.005	0.977	1.034
All ages and sex	NO_2 SO_2 O_3	1.029	0.991	1.069	1.032	1.01	1.055
Age 15 - 64		1.017	0.963	1.073	1.024	0.988	1.061
Age >65		1.04	0.988	1.096	1.041	1.013	1.07
Female		0.999	0.945	1.057	1.042	1.011	1.073
Male		1.055	1.004	1.108	1.025	0.994	1.058
All ages and sex	NO_2 PM_{10} SO_2 O_3	1.037	0.994	1.082	1.02	0.995	1.046
Age 15 - 64		1.016	0.957	1.079	1.029	0.988	1.072
Age >65		1.057	0.997	1.12	1.018	0.986	1.05
Female		1.011	0.949	1.077	1.026	0.992	1.062
Male		1.061	1.004	1.121	1.017	0.981	1.053

Table S8. Single and multiple pollutant model adjusted relative risk (RR) for 10 μg/m³ increase in the two-day moving average of SO_2 concentrations and mortality due to cardiovascular and respiratory diseases in all ages, age groups and sex, Cape Town, South Africa, 1 January 2006 - 31 December 2015.

		Respiratory disease			Cardiovascular disease		
	Pollutant (10 μg/m³)	Relative risk	95% Confidence interval		Relative risk	95% Confidence interval	
			Lower interval	Upper interval		Lower interval	Upper interval
All ages and sex	SO_2	1.021	0.984	1.06	1.023	1.001	1.047
Age 15 - 64		1.017	0.965	1.071	1.016	0.979	1.054
Age >65		1.026	0.975	1.08	1.028	0.999	1.057
Female		1.036	0.981	1.094	1.012	0.982	1.044
Male		1.009	0.96	1.06	1.037	1.004	1.07
All ages and sex	SO_2 PM_{10}	1.022	0.983	1.063	1.013	0.989	1.037
Age 15 - 64		1.018	0.964	1.076	1.012	0.973	1.051
Age >65		1.025	0.972	1.082	1.013	0.983	1.043
Female		1.033	0.975	1.094	0.997	0.966	1.03
Male		1.012	0.961	1.065	1.031	0.997	1.066
All ages and sex	SO_2 NO_2	1.003	0.962	1.046	1.016	0.992	1.042
Age 15 - 64		0.995	0.939	1.056	1.02	0.98	1.062
Age >65		1.012	0.956	1.071	1.013	0.983	1.045
Female		1.021	0.961	1.085	1.006	0.973	1.04
Male		0.987	0.934	1.042	1.029	0.994	1.065
All ages and sex	SO_2 O_3	1.003	0.954	1.056	1.011	0.981	1.043
Age 15 - 64		1.011	0.942	1.085	0.999	0.951	1.05
Age >65		0.999	0.931	1.072	1.02	0.981	1.06
Female		1.006	0.933	1.085	1.005	0.963	1.048
Male		1	0.936	1.068	1.021	0.977	1.066
All ages and sex	SO_2 PM_{10} O_3	1.007	0.955	1.063	0.994	0.962	1.027
Age 15 - 64		1.011	0.939	1.089	0.998	0.947	1.051

Age >65		1.005	0.933	1.082	0.992	0.952	1.034
Female		1.014	0.936	1.098	0.979	0.936	1.024
Male		1.002	0.935	1.074	1.012	0.966	1.06
All ages and sex	SO_2 PM_{10} NO_2	1.009	0.967	1.053	1.012	0.986	1.037
Age 15 - 64		1.002	0.944	1.064	1.021	0.98	1.063
Age >65		1.016	0.958	1.076	1.005	0.973	1.037
Female		1.027	0.965	1.093	0.997	0.964	1.032
Male		0.993	0.939	1.05	1.028	0.993	1.065
All ages and sex	SO_2 NO_2 O_3	0.992	0.937	1.05	0.995	0.962	1.03
Age 15 - 64		0.994	0.917	1.077	1.006	0.952	1.063
Age >65		0.994	0.918	1.076	0.988	0.945	1.032
Female		1.018	0.934	1.109	0.985	0.94	1.033
Male		0.972	0.902	1.047	1.007	0.959	1.057
All ages and sex	SO_2 PM_{10} NO_2 O_3	0.999	0.942	1.06	0.988	0.954	1.023
Age 15 - 64		0.997	0.919	1.083	1.015	0.959	1.074
Age >65		1.004	0.925	1.09	0.971	0.928	1.016
Female		1.026	0.939	1.12	0.973	0.927	1.021
Male		0.98	0.908	1.058	1.006	0.957	1.057

Table S9. Single and multiple pollutant model adjusted relative risk (RR) for 10 µg/m^3 increase in the two-day moving average of O_3 concentrations and mortality due to cardiovascular and respiratory diseases in all ages, age groups and sex, Cape Town, South Africa, 1 January 2006 - 31 December 2015.

		Respiratory disease			Cardiovascular disease		
	Pollutant (10 µg/m^3)	Relative risk	95% Confidence interval		Relative risk	95% Confidence interval	
			Lower interval	Upper interval		Lower interval	Upper interval
All ages and sex	O_3	1.004	0.978	1.031	1.016	1.001	1.031
Age 15 - 64		1.004	0.968	1.042	1.012	0.988	1.036
Age >65		1.003	0.967	1.04	1.019	1	1.037
Female		0.967	0.93	1.005	1.011	0.991	1.032
Male		1.034	0.999	1.071	1.022	1.001	1.043
All ages and sex	O_3 PM_{10}	1.002	0.975	1.029	1.016	1.001	1.032
Age 15 - 64		1.003	0.966	1.042	1.013	0.988	1.038
Age >65		1	0.963	1.038	1.02	1	1.04
Female		0.964	0.926	1.003	1.015	0.994	1.037
Male		1.032	0.996	1.07	1.019	0.997	1.042
All ages and sex	O_3 NO_2	0.999	0.971	1.028	1.014	1.002	1.027
Age 15 - 64		0.993	0.954	1.033	1.016	0.996	1.038
Age >65		1.004	0.965	1.044	1.014	0.998	1.031
Female		0.956	0.917	0.997	1.013	0.995	1.03
Male		1.034	0.996	1.074	1.018	1	1.037
All ages and sex	O_3 SO_2	1.004	0.977	1.031	1.014	0.999	1.029
Age 15 - 64		1.003	0.967	1.041	1.009	0.985	1.033
Age >65		1.004	0.967	1.041	1.017	0.999	1.037
Female		0.969	0.932	1.008	1.01	0.989	1.03
Male		1.032	0.997	1.07	1.02	0.999	1.042
All ages and sex	O_3 PM_{10} SO_2	1.001	0.974	1.029	1.017	1.002	1.032
Age 15 - 64		1.001	0.963	1.04	1.009	0.985	1.034

Age >65		1.001	0.964	1.04	1.023	1.004	1.042
Female		0.966	0.928	1.005	1.014	0.994	1.035
Male		1.03	0.993	1.068	1.022	1.001	1.044
All ages and sex	O_3 PM_{10} NO_2	0.995	0.967	1.024	1.017	1.002	1.033
Age 15 - 64		0.989	0.95	1.03	1.012	0.986	1.037
Age >65		1	0.96	1.041	1.022	1.002	1.042
Female		0.953	0.913	0.994	1.017	0.996	1.038
Male		1.029	0.991	1.07	1.019	0.997	1.042
All ages and sex	O_3 SO_2 NO_2	0.999	0.971	1.028	1.016	1.001	1.032
Age 15 - 64		0.993	0.954	1.033	1.012	0.987	1.037
Age >65		1.004	0.965	1.045	1.02	1.001	1.04
Female		0.957	0.918	0.998	1.014	0.993	1.035
Male		1.034	0.995	1.074	1.021	0.999	1.043
All ages and sex	O_3 PM_{10} NO_2 SO_2	0.995	0.967	1.025	1.017	1.002	1.033
Age 15 - 64		0.989	0.949	1.03	1.01	0.985	1.036
Age >65		1	0.96	1.041	1.023	1.003	1.043
Female		0.953	0.914	0.995	1.016	0.995	1.038
Male		1.029	0.99	1.07	1.021	0.999	1.043

Table S10. Warm and dry—Season-specific single and multiple pollutant model adjusted relative risk (RR) for 10 µg/m^3 increase in the two-day moving average of PM_{10}, NO_2, SO_2 and O_3 concentrations and mortality due to cardiovascular and respiratory diseases in all ages, Cape Town, South Africa, 1 January 2006 - 31 December 2015.

		Respiratory disease			Cardiovascular disease		
	Pollutant (10 µg/m^3)	Relative risk	95% Confidence interval		Relative risk	95% Confidence interval	
			Lower interval	Upper interval		Lower interval	Upper interval
All ages and sex	PM_{10}	1.01	0.987	1.033	1.003	0.99	1.016
	NO_2	1.058	1.011	1.107	1.004	0.979	1.03
	SO_2	1.011	0.953	1.072	0.991	0.959	1.023
	O_3	1.02	0.984	1.056	1.002	0.984	1.02
All ages and sex							
	PM_{10} NO_2 SO_2 O_3	1.008	0.966	1.053	1.006	0.982	1.031
	NO_2 PM_{10} SO_2 O_3	1.134	1.044	1.233	1.019	0.972	1.068
	SO_2 PM_{10} NO_2 O_3	0.967	0.876	1.068	0.995	0.94	1.052
	O_3 PM_{10} NO_2 SO_2	0.969	0.922	1.019	1.016	0.991	1.042

Table S11. Cold and wet - Season-specific single and multiple pollutant model adjusted relative risk (RR) for 10 µg/m³ increase in the two-day moving average of PM_{10}, NO_2, SO_2 and O_3 concentrations and mortality due to cardiovascular and respiratory diseases in all ages, Cape Town, South Africa, 1 January 2006 - 31 December 2015.

		Respiratory disease			Cardiovascular disease		
	Pollutant (10 µg/m³)	Relative risk	95% Confidence interval		Relative risk	95% Confidence interval	
			Lower interval	Upper interval		Lower interval	Upper interval
All ages and sex	PM_{10}	1	0.984	1.016	1.016	1.007	1.025
	NO_2	1.039	1.01	1.07	1.024	1.006	1.041
	SO_2	1.024	0.985	1.064	1.034	1.01	1.059
	O_3	0.999	0.972	1.027	1.019	1.004	1.034
All ages and sex							
	PM_{10} NO_2 SO_2 O_3	0.991	0.964	1.019	1.015	0.998	1.032
	NO_2 PM_{10} SO_2 O_3	1.021	0.973	1.071	1.017	0.988	1.047
	SO_2 PM_{10} NO_2 O_3	1.012	0.943	1.086	0.985	0.943	1.028
	O_3 PM_{10} NO_2 SO_2	1	0.97	1.032	1.018	1.001	1.035

Table S12. Warm and dry - Season-specific single and multiple pollutant model adjusted relative risk (RR) for an interquartile range increase in the two-day moving average of PM_{10}, NO_2, SO_2 and O_3 concentrations and mortality due to cardiovascular and respiratory diseases in all ages, Cape Town, South Africa, 1 January 2006 - 31 December 2015.

		Respiratory disease			Cardiovascular disease		
	Pollutant (10 µg/m³)	Relative risk	95% Confidence interval		Relative risk	95% Confidence interval	
			Lower interval	Upper interval		Lower interval	Upper interval
All ages and sex	PM_{10}	1.016	0.978	1.054	1.004	0.984	1.026
	NO_2	1.062	1.012	1.114	1.004	0.978	1.032
	SO_2	1.006	0.972	1.042	0.994	0.975	1.014
	O_3	1.031	0.976	1.089	1.003	0.976	1.032
All ages and sex							
	PM_{10} NO_2 SO_2 O_3	1.013	0.944	1.087	1.011	0.979	1.044
	NO_2 PM_{10} SO_2 O_3	1.144	1.047	1.25	1.011	0.978	1.044

	SO_2 PM_{10} NO_2 O_3	0.98	0.924	1.04	0.999	0.974	1.025
	O_3 PM_{10} NO_2 SO_2	0.952	0.881	1.029	1.028	0.999	1.057

Table S13. Cold and wet - Season-specific single and multiple pollutant model adjusted relative risk (RR) for an interquartile range increase in the two-day moving average of PM_{10}, NO_2, SO_2 and O_3 concentrations and mortality due to cardiovascular and respiratory diseases in all ages, Cape Town, South Africa, 1 January 2006 - 31 December 2015.

		Respiratory disease			Cardiovascular disease		
	Pollutant (10 μg/m^3)	Relative risk	95% Confidence interval		Relative risk	95% Confidence interval	
			Lower interval	Upper interval		Lower interval	Upper interval
All ages and sex	PM_{10}	1	0.975	1.026	1.026	1.011	1.042
	NO_2	1.042	1.011	1.074	1.025	1.007	1.044
	SO_2	1.014	0.991	1.038	1.02	1.006	1.035
	O_3	0.999	0.957	1.042	1.029	1.006	1.053
All ages and sex							
	PM_{10} NO_2 SO_2 O_3	0.986	0.942	1.031	1.023	0.99	1.058
	NO_2 PM_{10} SO_2 O_3	1.022	0.971	1.076	1.032	0.992	1.073
	SO_2 PM_{10} NO_2 O_3	1.007	0.965	1.051	0.986	0.955	1.019
	O_3 PM_{10} NO_2 SO_2	1.001	0.954	1.05	1.036	1.006	1.067

Lag structures

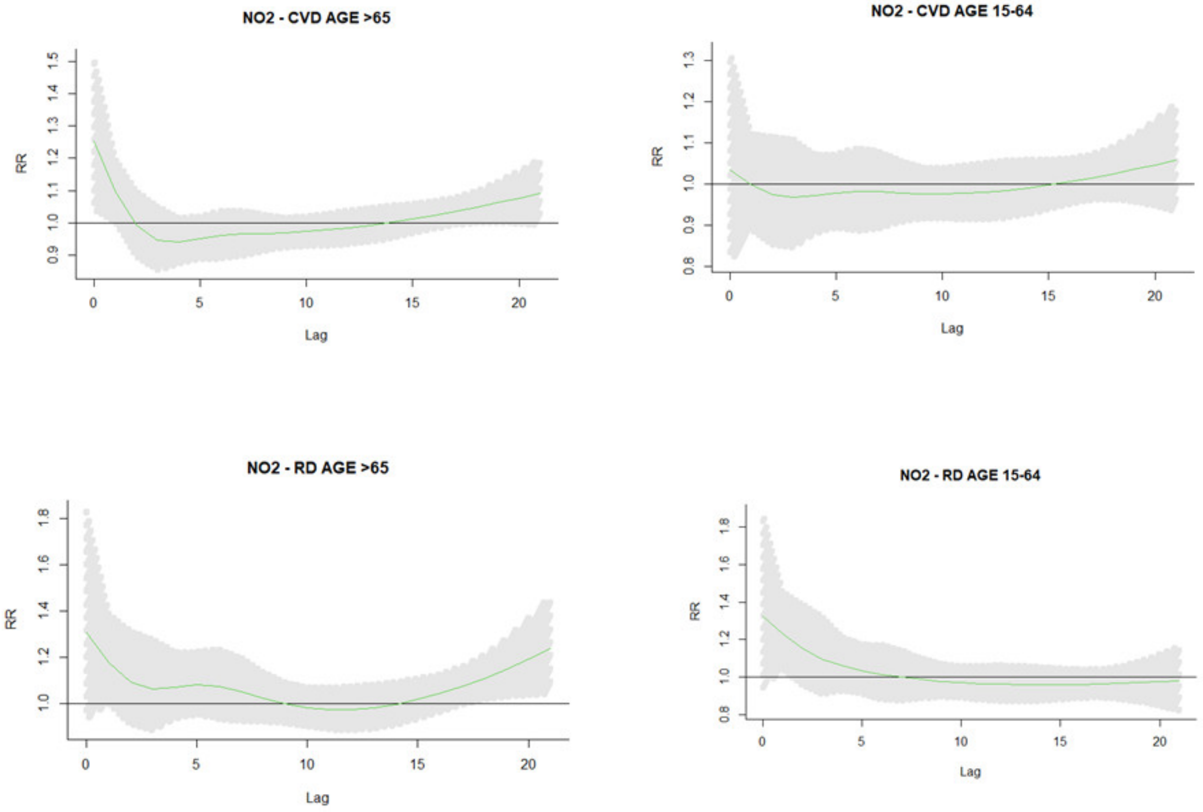

Figure S1. Age-specific Lag structure (0–21) of the estimated effects of a 10 μg/m³ increase in NO_2 concentrations on cardiovascular and respiratory disease mortality in Cape Town, South Africa, 2006–2015. The green curve gives the RR-estimates and the light grey band their 95% confidence intervals.

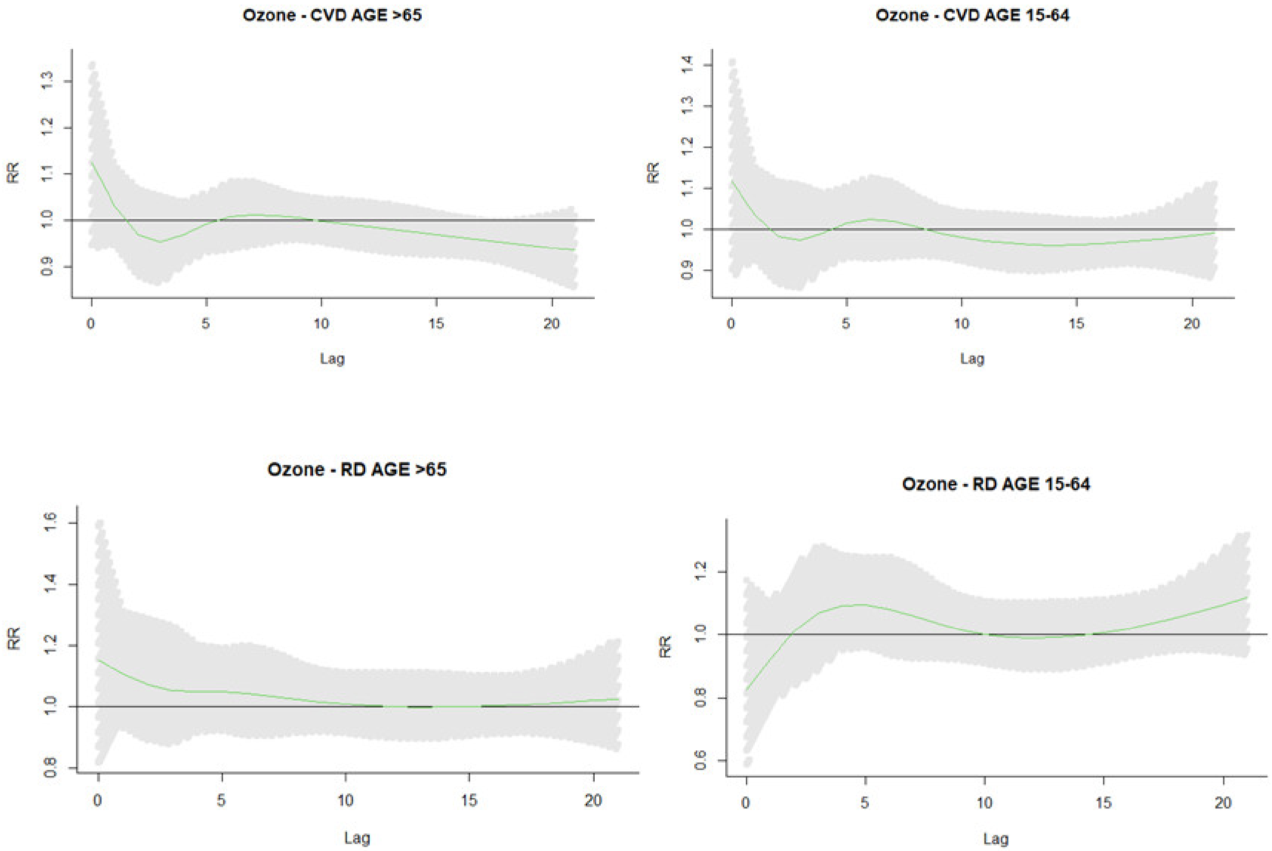

Figure S2. Age-Specific lag structure (0–21) of the estimated effects of a 10 μg/m³ increase in O_3 concentrations on cardiovascular and respiratory disease mortality in Cape Town, South Africa, 2006–2015. The green curve gives the RR-estimates and the light grey band their 95% confidence intervals.

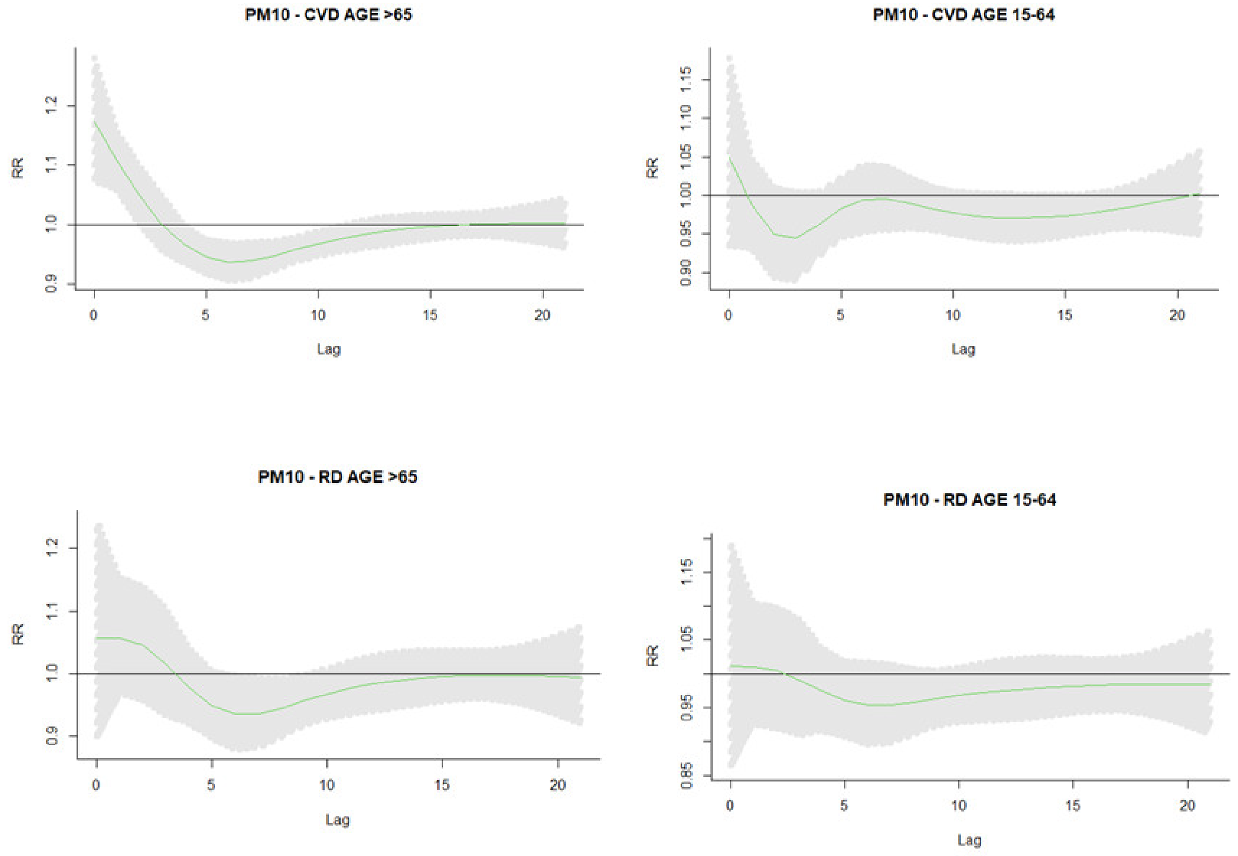

Figure S3i. Age-Specific lag structure (0–21) of the estimated effects of a 10 μg/m3 increase in PM_{10} concentrations on cardiovascular and respiratory disease mortality in Cape Town, South Africa, 2006–2015. The green curve gives the RR-estimates and the light grey band their 95% confidence intervals

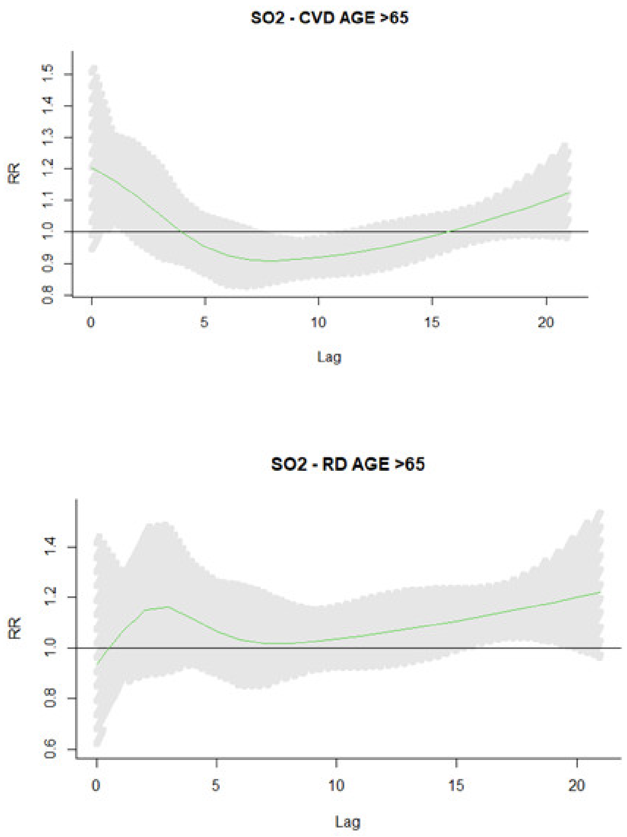

Figure S4. Age-Specific lag structure (0–21) of the estimated effects of a 10 µg/m3 increase in SO_2 concentrations on cardiovascular and respiratory disease mortality in Cape Town, South Africa, 2006–2015. The green curve gives the RR-estimates and the light grey band their 95% confidence intervals.

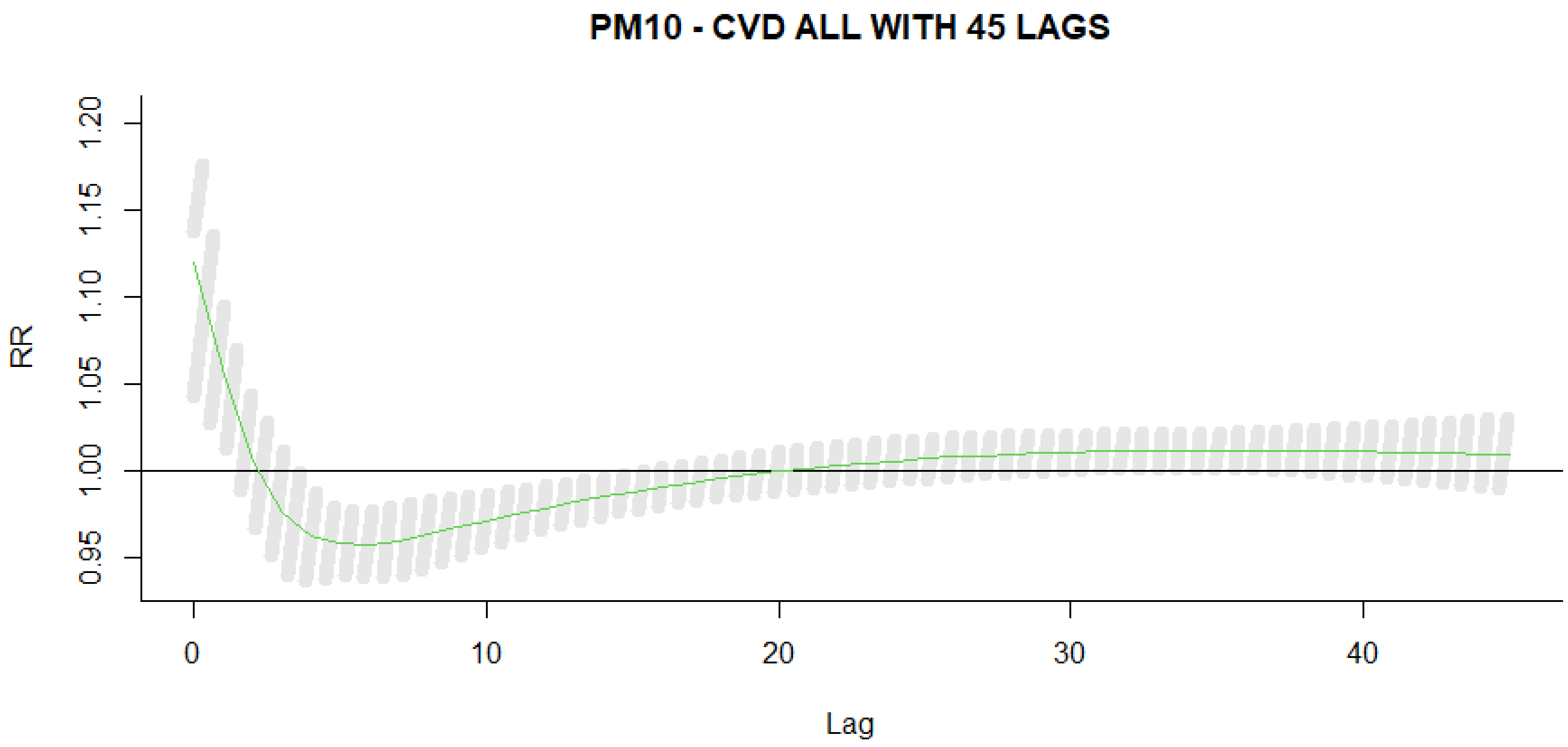

Figure S5. Overall lag structure (0–45) of the estimated effects of a 10 μg/m³ increase in PM_{10} concentrations on cardiovascular disease mortality in Cape Town, South Africa, 2006–2015. The green curve gives the RR-estimates and the light grey band their 95% confidence intervals.

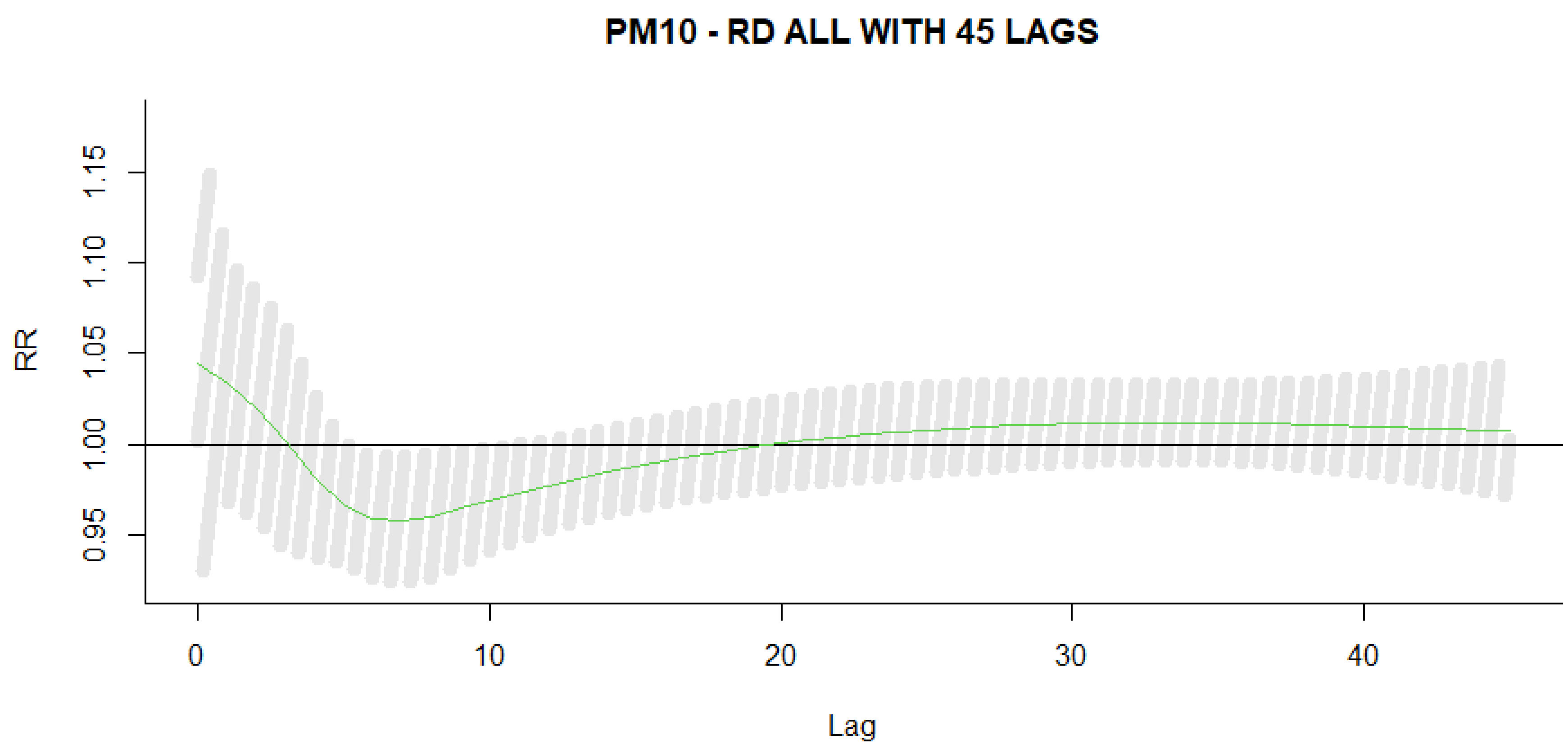

Figure S6. Overall lag structure (0–45) of the estimated effects of a 10 μg/m³ increase in PM_{10} concentrations on respiratory disease mortality in Cape Town, South Africa, 2006–2015. The green curve gives the RR-estimates and the light grey band their 95% confidence intervals.
